# Organize and mobilize for implementation effectiveness to improve overdose education and naloxone distribution from syringe services programs: a randomized controlled trial

**DOI:** 10.1186/s13012-024-01354-y

**Published:** 2024-02-28

**Authors:** Barrot H. Lambdin, Ricky N. Bluthenthal, Bryan R. Garner, Lynn D. Wenger, Erica N. Browne, Terry Morris, Lee Ongais, Cariné E. Megerian, Alex H. Kral

**Affiliations:** 1 RTI International, 2150 Shattuck Avenue, 8Th Floor, Berkeley, CA 94704 USA; 2 Department of Epidemiology and Biostatistics, University of California San Francisco, San Francisco, CA USA; 3 Department of Global Health, University of Washington, Seattle, WA USA; 4 Keck Medicine, Department of Population and Public Health Sciences, University of Southern California, 1975 Zonal Ave, Los Angeles, CA 90033 USA; 5 Department of Internal Medicine, College of Medicine, The Ohio State University, 370 W. 9Th Avenue, Columbus, OH 43210 USA; 6 San Francisco AIDS Foundation, 1035 Market Street, 4Th Floor, San Francisco, CA 94103 USA

**Keywords:** Overdose education and naloxone distribution, Syringe services programs, Implementation strategy, Implementation science

## Abstract

**Background:**

The United States (US) continues to face decades-long increases in opioid overdose fatalities. As an opioid overdose reversal medication, naloxone can dramatically reduce opioid overdose mortality rates when distributed to people likely to experience or witness an opioid overdose and packaged with education on its use, known as overdose education and naloxone distribution (OEND). Syringe services programs (SSPs) are ideal venues for OEND with staff who are culturally competent in providing services for people who are at risk of experiencing or observing an opioid overdose. We carried out a randomized controlled trial of SSPs to understand the effectiveness of the organize and mobilize for implementation effectiveness (OMIE) approach at improving OEND implementation effectiveness within SSPs.

**Methods:**

Using simple randomization, 105 SSPs were enrolled into the trial and assigned to one of two study arms — (1) dissemination of OEND best practice recommendations (Control SSPs) or the OMIE approach along with dissemination of the OEND best practice recommendations (i.e., OMIE SSPs). OMIE SSPs could participate in 60-min OMIE sessions once a month for up to 12 months. At 12-month post-baseline, 102 of 105 SSPs (97%) responded to the follow-up survey.

**Results:**

The median number of sessions completed by OMIE SSPs was 10. Comparing OMIE SSPs to control SSPs, we observed significant increases in the number of participants receiving naloxone (incidence rate ratio: 2.15; 95% *CI*: 1.42, 3.25; *p* < 0.01) and the rate of naloxone doses distributed per SSP participant (adjusted incidence rate ratio: 1.97; 95% *CI*: 1.18, 3.30; *p* = 0.01). We observed no statistically significant difference in the number of adopted best practices between conditions (difference in means 0.2, 95% *CI*: − 0.7, 1.0; *p* = 0.68). We also observed a threshold effect where SSPs receiving a higher OMIE dose had greater effect sizes with regard to the number of people given naloxone and the number of naloxone doses distributed.

**Conclusions:**

In conclusion, the multifaceted OMIE approach was effective at increasing naloxone distribution from SSPs, despite substantial external shocks during the trial. These findings have major implications for addressing the overdose crisis, which has continued unabated for decades.

**Trial registration:**

ClinicalTrials.gov, NCT03924505. Registered 19 April 2019.

**Supplementary Information:**

The online version contains supplementary material available at 10.1186/s13012-024-01354-y.

Contributions to the literature

- Despite decades-long increases in opioid overdose fatalities, no implementation science trials have tested whether implementation strategies could advance implementation effectiveness of naloxone within community-based settings.
- The multicomponent organize and mobilize for implementation effectiveness (OMIE) approach effectively increased naloxone distribution from syringe services programs.
- These findings show that facilitation-based approaches, like OMIE, were successful even in the context of major external shocks (i.e., COVID-19).
- Our findings have major implications for addressing opioid overdose throughout the nation, bringing advancements in implementation science approaches to a decades-long health crisis.

## Background

The United States (US) continues to face decades-long increases in opioid overdose mortality [1]. The Centers for Disease Control and Prevention (CDC) estimates nearly 775,000 people have died of an opioid overdose since 1999 [2]. Between 2020 and 2021, overdose mortality rates involving heroin and synthetic opioids, other than methadone, increased 22%. Overall, drug overdose deaths in the US increased for all genders; all age groups over the age of 25; American Indian/Alaskan Native, non-Hispanic White, non-Hispanic Black, Hispanic, and Native Hawaiian/Pacific Islander communities; all census regions; and urban, suburban, and rural settings [3].

Opioid overdoses involve a toxic dose of natural opioids like heroin, semisynthetic opioids like oxycodone, and/or synthetic opioids like fentanyl. Naloxone, an opioid antagonist, is an evidence-based biomedical intervention that effectively reverses opioid overdose when administered intranasally or intramuscularly [4–7]. Opioid antagonists bind to the brain’s opioid receptor sites, which displaces any opioid currently in the system and temporarily negates their effects, saving the individual’s life. Use of naloxone does not cause dependence or tolerance and can, though not always, precipitate withdrawal [5–8]. Naloxone can dramatically reduce opioid overdose mortality when distributed to people likely to experience or witness an opioid overdose and packaged with education on its use, known as overdose education and naloxone distribution (OEND) [9–14].

Syringe services programs (SSPs) have a primary function of distributing safe drug use supplies, such as sterile syringes, pipes, and injection-related equipment, to facilitate the reduction of harms associated with drug use for their participants. Often, SSPs are the first to hear from their participants regarding changes in the unregulated drug market, drug use trends, and overdose experiences. Most SSPs provide a variety of other evidence-based interventions to improve the health of people who use drugs, including OEND [15–18]. SSPs reach people at high risk for experiencing or witnessing an opioid overdose and pioneered the development of OEND [19–21]. With staff who are culturally competent in providing services for people who use opioids and preexisting delivery systems designed to reach participants in their own environment, SSPs are an ideal venue for OEND.

Research documenting OEND’s effectiveness (i.e., intervention effectiveness) for reducing opioid overdose mortality emerged over 15 years ago [7]. Only recently have studies begun to assess elements of OEND implementation effectiveness within SSPs [22–26]. As an organizational-level construct, implementation effectiveness can be defined as the aggregated consistency and quality of intervention use within an organization [27]. Findings from recent research identify factors from both the external and internal context of SSPs that shape effective implementation of OEND [22–26]. Yet, to date, no studies have assessed whether implementation strategies (i.e., approaches to improve the adoption, implementation, or sustainment of evidence-based practices) can advance implementation effectiveness of OEND within SSPs.

External facilitation-based implementation strategies have been identified as a promising approach to advance implementation outcomes [28–39]. Fundamentally, these approaches involve a person external to the organization providing interactive problem-solving and support to assist implementation efforts. Often, their work occurs in conjunction with other implementation strategies. Whether such an approach can help SSPs, which often face substantial community, financial, and legal constraints [40], effectively implement OEND remains an important area of inquiry. As such, we conducted a randomized controlled trial of 105 SSPs throughout the US to understand the effectiveness of a multifaceted, external facilitation-based implementation strategy at advancing OEND implementation effectiveness within SSPs.

## Methods

### Trial design

We conducted a randomized controlled trial of SSPs throughout the US and US territories. SSPs were assigned to one of two implementation conditions using simple randomization. A standardized checklist aided this paper’s clarity and transparency for describing an intervention and reporting a randomized controlled trial [41, 42]. RTI International’s Institutional Review Board (IRB) approved and provided oversight for all research activities (STUDY00020448). This trial was registered at ClinicalTrials.gov as NCT03924505.

### Study setting

The target population included operators of SSPs located throughout the US and US territories. Prior to launching the trial, 342 SSPs were known to be operating in the US and US territories. Described elsewhere [25], our team launched a national survey of syringe services programs (NSSSP) in February 2019 of all known SSPs operating throughout the US and its territories, receiving a response from 263 (77%) SSPs. NSSSP responding SSPs were located in the Northeast (13%), Midwest (21%), South (24%), and West (42%) census regions. Among the responding SSPs, 247 (94%) were implementing OEND for their participants [25].

### Recruitment, eligibility, and enrollment

To be eligible, an organization must have the following: (1) met the definition of an SSP — a program which primary function is to engage people who use drugs and provide them free drug use supplies to reduce harms associated with drug use, (2) implemented OEND for a minimum of 6 months, and (3) completed the NSSSP fielded from February to July 2019. We excluded organizations such as fire departments or emergency departments of hospitals that distributed drug use supplies since it would be an ancillary function of these organizations and OEND programs that were not part of a SSP.

A total of 243 SSPs participated in the survey and were determined to be eligible. SSPs were organized into a randomized list and were contacted sequentially for recruitment into the trial. We recruited SSPs from September 2019 to February 2021. Within that timeframe, we paused recruitment activities from March to July 2020 due to the onset of the COVID-19 pandemic. The pause in recruitment activities primarily provided an opportunity for SSPs to adapt to COVID-19 changes, particularly for their adjustment to stay-at-home orders and adoption of delivery models that minimized close person-to-person contact.

To recruit SSPs, our study team initially contacted people in leadership role(s) at each SSP via email to set up a call to explain the study and carry out enrollment activities for organizations that were interested. Organizational leadership included executive directors, program managers, and/or site coordinators. For organizations interested in participating in the trial, study staff would obtain electronic/written informed consent from the organization to join the study, carry out the organizational agreement for participation, and administer the baseline survey. Following the baseline survey, SSPs were randomized using simple randomization to two study arms: (1) dissemination of OEND best practice recommendations (i.e., Control SSPs) or (2) the organize and mobilize for implementation effectiveness (OMIE) approach along with dissemination of the OEND best practice recommendations (i.e., OMIE SSPs). Sequentially, numbered sealed envelopes were used to implement the random allocation sequence, which were concealed until the point of randomization.

### OEND best practice recommendations

Study staff disseminated best practice recommendations to all SSPs enrolled in the trial. Details of OEND best practice recommendations can be found elsewhere [43]. Briefly, a Delphi study was carried out to develop a set of best practices for OEND implementation within SSPs. Experts for the Delphi study included people in paid and volunteer leadership and direct service positions in SSPs, OEND researchers, people who work in state or local health departments, and people who use drugs who deliver and access SSP/OEND services. All individuals had prior or current experience delivering OEND programming in community-based settings, and people with lived and living substance use experience were represented in each of the expert categories. Findings from this initiative were summarized into a best practices implementation guide (Additional file 1: Appendix A).

### Organize and mobilize for implementation effectiveness (OMIE)

Table 1 defines and specifies the eight discrete implementation strategies that comprised OMIE. The OMIE approach was based on the implementation and sustainment facilitation (ISF) strategy, which is grounded in the theory of implementation effectiveness [27] and added discrete strategies from the Addiction Technology Transfer Center (ATTC), which were considered necessary elements from the original trial. Both ISF and ATTC have been described extensively elsewhere [44, 45]. Overall, our multicomponent approach used external facilitation as the overarching strategy, by which seven other strategies were leveraged to support SSP staff and leadership. In total, 4 out of 7 discrete strategies from ISF were combined with 4 out of 10 discrete strategies from ATTC, detailed in Table 1. In addition to external facilitation, our multifaceted OMIE approach included the following: organize implementation team meetings, identify and prepare champions, develop and organize quality monitoring system, assess for readiness and identify barriers, distribute educational materials and resources, conduct educational meetings, and provide ongoing consultation. The study team comprised of people who had delivered SSP-based OEND services in the past year, researchers with over 20 years of SSP and OEND research experience, and national OEND implementation experts, who collaboratively decided upon the discrete strategies for the OMIE approach. Decisions were grounded in shared understanding and discussion of implementation barriers SSPs faced generally and with OEND specifically.

**Table 1:** Specification of organize and mobilize for implementation effectiveness

Discrete strategy	Definition, actor, action, action target, and temporality specification
1. External facilitation	*Definition*: Provision of guidance, resources, and coaching from an implementation expert who is external to the organization *Actor*: Individual with previous OEND implementation experience within SSP settings *Action*: Overarching mechanism by which the below strategies were delivered to SSP staff *Action target*: SSP staff, including naloxone implementation team and leadership *Temporality*: Begins within 1 month of enrolling into the trial and occurs up to monthly for 12 months
2. Organize implementation team meetings	*Definition*: Develop and support implementation team who are implementing OEND, giving them protected time to focus on implementation efforts, share experiences, and support one another *Actor*: External implementation advisor (see above) *Action*: Web-based meetings with direct interaction between external implementation advisor and SSP staff *Action target*: SSP staff, including naloxone implementation team and leadership *Temporality*: Begins within 1 month of enrolling into the trial and occurs up to monthly for 12 months
3. Identify and prepare champions	*Definition*: Cultivate relationships with people who will champion and generate excitement within the organization regarding OEND best practice implementation *Actor*: External implementation advisor (see above) *Action*: Focused relationship building with SSP staff who are OEND implementation champion(s) *Action target*: SSP staff, including OEND implementation team and leadership *Temporality*: Begins with first organizational team meeting and continues as needed for 12 months
4. Develop and organize quality monitoring system	*Definition*: Develop and introduce an electronic tool that can be used to assess and prioritize best practice implementation efforts *Actor*: External implementation advisor (see above) *Action*: Using the electronic tool, external implementation advisor works with SSP staff to assess OEND best practice implementation and prioritize areas for focused efforts *Action target*: SSP staff, including OEND implementation team and leadership *Temporality*: Begins with first organizational team meeting and continues up to monthly for 12 months
5. Assess for readiness and identify barriers	*Definition*: Assess SSPs to determine degree of readiness for best practice implementation and barriers that may impede implementation *Actor*: External implementation advisor (see above) *Action*: Focused conversation to discuss activities required for best practice implementation, readiness to carry out those activities, and potential barriers that could impede those efforts *Action target*: SSP staff, including OEND implementation team and leadership *Temporality*: Begins within 1 month of enrolling into the trial and occurs up to monthly for 12 months
6. Distribute educational materials and resources	*Definition*: Distribute educational materials (manuals, online trainings, etc.) electronically with regard to OEND best practices *Actor*: External implementation advisor (see above) *Action*: Electronically distribute OEND best practices manual and other resources including implementation manuals and online trainings *Action target*: SSP staff, including OEND implementation team and leadership *Temporality*: Begins with first organizational team meeting and continues as needed for 12 months
7. Conduct educational meetings	*Definition*: Conduct educational sessions for providers and leadership with regard to OEND best practices *Actor*: External implementation advisor (see above) *Action*: Web-based trainings with direct interaction between external implementation advisor and SSP staff *Action target*: SSP staff, including OEND implementation team and leadership *Temporality*: Begins with first organizational team meeting and continues as needed for 12 months
8. Provide ongoing consultation	*Definition*: Provide implementers with continued consultation with regard to OEND best practices *Actor*: External implementation advisor (see above) *Action*: Web-based meetings with direct interaction between external implementation advisor and SSP staff *Action target*: SSP staff, including OEND implementation team and leadership *Temporality*: Begins within 1 month of enrolling into the trial and occurs via web-based meetings up to monthly for 12 months and as needed via electronic communication

SSP staff and organizational leadership in the OMIE arm were provided the opportunity to participate in 60-min sessions once a month for up to 12 months. In addition to monthly sessions, facilitators were provided up to 2 h to prepare for sessions and to identify and distribute resources to SSPs based on identified priorities. All sessions were offered virtually over audiovisual connections. Thus, the maximum possible dose for each SSP was 12 sessions or 36 h. To maximize the extent to which the approach was implemented with consistency and quality, the project’s lead and the study coordinator trained each facilitator, reviewed randomly selected facilitation session recordings, and held group supervisory meetings weekly to discuss successes, lessons learned, and emerging issues.

### Data collection

We carried out an interview-administered survey at SSPs baseline visit and at their 12-month follow-up after all facilitation-based activities had occurred. SSPs were paid a US $50 incentive for their time (~ 30 min) completing the baseline and the 12-month follow-up survey. In addition, the study coordinator tracked the number of sessions and the number of hours between external facilitators and SSP teams as part of a log of implementation activities. Follow-up to complete the 12-month follow-up survey ended in February 2022, marking the end of the trial approximately 12 months after the last SSP was enrolled.

### Trial outcomes

Our primary outcomes center on the multidimensional variable of implementation effectiveness (i.e., the consistency and quality of OEND implementation by SSP staff). Implementation effectiveness fit our broad objectives, by focusing on the extent to which our multifaceted, facilitation-based approach impacted OEND delivery at the organizational level. Accordingly, SSPs represent the primary unit of analysis. Proctor et al.’s taxonomy of implementation outcomes informs our operationalization of implementation effectiveness, focusing on the reach of SSPs’ naloxone distribution (i.e., consistency) and fidelity to OEND best practices (i.e., quality) [46]. As such, the primary outcomes were the number of naloxone doses distributed in the past 3 months; the number of SSP participants receiving naloxone in the past 3 months, and the number of OEND best practices implemented. Importantly, prior research has shown that larger scale naloxone distribution has led to reductions in opioid overdose mortality, further supporting use of these measures as trial outcomes [17, 47, 48].

### Covariates

The baseline survey also collected information on region of operation, the number of staff and volunteers at the SSP, staff training in OEND, the prior year’s annual budget in dollars, and the number of participant contacts at the SSP in the past 3 months.

### Masking

Masking the SSP organizations and their staff to the assigned implementation strategy condition was not possible. Aside from the external facilitators who delivered the implementation strategies, research staff were blinded to all condition assignments, including the statistician throughout intent-to-treat analyses.

### Statistical methods

Statistical analyses were conducted using an intent-to-treat approach. Descriptive statistics were used to summarize continuous outcomes and covariates by experimental condition; frequencies and percentages were used to summarize categorical measures. Given the varying sizes of SSPs, counts of naloxone doses and individuals receiving naloxone were summarized as a rate per number of participant contacts for syringe services in the past 3 months.

Negative binomial regression models were used to compare the number of naloxone doses distributed and contacts for naloxone refills or trainings by condition; the number of contacts for syringe services in the past 3 months was included as an offset. The model for naloxone doses was adjusted for baseline rate given the difference between conditions at baseline. The mean number of best practices adopted was compared by condition using a two-sample *t*-test. We also carried out a per-protocol dose response analyses, where OMIE SSPs were dichotomized at the median for the number of OMIE sessions (< 10 sessions; 10 + sessions) and number of OMIE hours received (< 12 h; 12 + h) and compared to Control SSPs. *p*-values < 0.05 were considered statistically significant a priori. All analyses were conducted using Stata 16.1 (StataCorp LLC, TX, USA).

### Targeted sample size

The targeted sample size was estimated using PASS software [49]. We estimated that a sample of 100 SSPs (50 per arm) would provide 80% statistical power to detect a statistically significant (*p* < 0.05) medium effect size (Cohen’s *d* = 0.45) [50] between study arms, assuming an alpha of 0.05, standard deviation of 1.0, and two repeated measurements (baseline and 12-month follow-up) with a first-order autoregressive covariance structure and a correlation between observations on the same SSP of 0.3.

## Results

### SSP participant flow

**Fig. 1:**
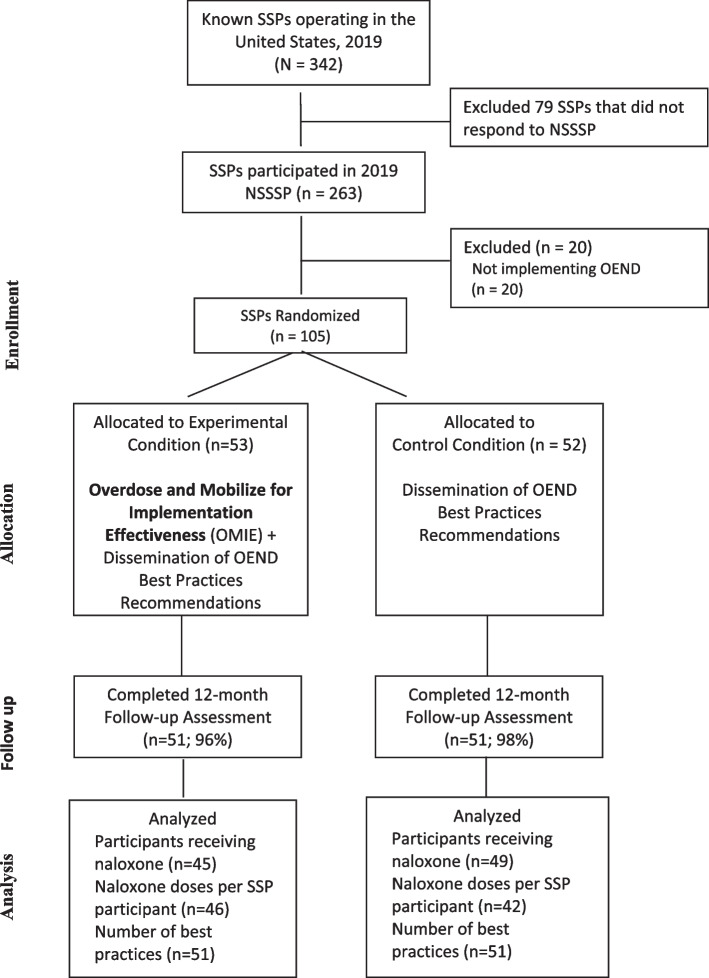
Consort flow diagram of syringe services programs enrolling into OMIE trial

A total of 105 SSPs enrolled and completed the baseline survey — 53 of which were randomized to the experimental condition (OMIE SSPs) and 52 of which were randomized to the control condition (control SSPs). At the 12-month follow-up visit, 102 of 105 SSPs (97%) responded to the follow-up survey — 51 (96%) from the experimental condition and 51 (98%) from the control condition. SSPs (*n* = 3) lost to follow-up did not respond to the study team’s requests to complete the 12-month follow-up survey. Regarding our study outcomes, 88 SSPs provided baseline and follow-up data about the number of SSP contacts and the number of naloxone doses distributed, 94 SSPs reported follow-up data about the number of SSP contacts and the number of people receiving naloxone, and 102 SSPs provided follow-up data for number of OEND best practices implemented (Fig. 1).

### Baseline characteristics

SSPs were well dispersed across the US and ranged in size; SSPs had a median of 7 staff and/or volunteers and a median annual budget of about US $120,000 (Table 2). At enrollment, SSPs reported implementing an average of 12 of 19 OEND best practices (range 5–17). In the previous 3 months, they had a mean of approximately 1500 contacts for services (standard deviation [SD] 1742) and distributed 1.7 naloxone doses per SSP contact (*SD* 2.5). The average number of naloxone doses distributed per contact was higher among OMIE SSPs (2.2), compared to Control SSPs (1.2) at baseline: incident rate ratio [IRR] 1.8, 95% *CI*: 1.2, 2.8, and *p* = 0.007. About one of every two contacts for services received naloxone training and/or refills (*SD* 0.73).

**Table 2:** Baseline characteristics of syringe services programs by study arm (*N*** = **105)

	**Control SSPs** **(*n* = 52)**	**OMIE SSPs** **(*n* = 53)**	**Total SSPs (*N* = 105)**
**Census region**, *n* (%)			
West	24 (47)	25 (47)	49 (47)
Midwest	12 (23)	12 (23)	24 (23)
South	13 (25)	8 (15)	21 (20)
North	1 (2)	8 (15)	9 (9)
Puerto Rico	2 (4)	0 (0)	2 (2)
**Number of staff & volunteers**, median (IQR)	7 (3.14)	7 (4.13)	7 (4.13)
**All staff can provide OEND**, *n* (%)	34 (65)	30 (57)	64 (61)
**Annual budget**, median (IQR)	US $106,291 (24,125–240,000)	US $145,500 (29,785–384,000)	US $119,864 (25,000–300,000)
**SSP participant contacts**^a^, mean (sd)	1422 (1821)	1623 (1671)	1522 (1742)
**Naloxone doses per contact**^a^, mean (sd)	1.2 (1.4)	2.2 (3.1)	1.7 (2.5)
**Participants receiving naloxone per contact**, mean (sd)	0.46 (0.51)	0.62 (0.91)	0.54 (0.73)
**Priority to expand naloxone staff**	82 (22)	85 (22)	84 (22)
**Priority to expand naloxone leadership**	86 (18)	84 (24)	85 (21)
**Number of OEND best practices implemented (19 items), mean**	12.2	12.0	12.1
Staff training and support (6 items)	2.5	2.6	2.5
Naloxone saturation and supply (4 items)	2.8	2.7	2.8
Culturally appropriate service (4 items)	2.7	2.5	2.6
Grounded in harm reduction (5 items)	4.3	4.2	4.2
**Sessions**, median (IQR)		10 (8–11)	
**Hours**, median (IQR)		12 (8–13)	

*OEND* Overdose education and naloxone distribution, *sd* Standard deviation

^a^Past 3 months

### Dose

Over the year following the baseline survey, the median dose received by SSPs, as measured by the number of sessions completed by OMIE SSPs, was 10 (*IQR*: 8–11). The median dose as measured by the number of OMIE support hours received by SSPs was 12 h (*IQR*: 8.3–13.5).

### Trial outcomes

In our intent-to-treat analysis, the average number of SSP participants that received naloxone from OMIE SSPs in the past 3 months was 2.15 times the rate among Control SSPs (95% *CI*: 1.42, 3.25; *p* < 0.01; Table 3). Additionally, controlling for baseline distribution rate, the rate of naloxone doses distributed per SSP participant contact among OMIE SSPs was 1.97 times the distribution rate among Control SSPs (95% *CI*: 1.18, 3.30; *p* = 0.01). On average among OMIE SSPs, 4 naloxone doses were distributed per contact, compared to 1.3 among Control SSPs, and 0.9 individuals per contact among OMIE SSPs received naloxone training or refills, compared to 0.4 among Control SSPs. At follow-up, SSPs had implemented 13 OEND best practices, on average. We observed no statistically significant difference in the number of adopted best practices between study arms (difference in means 0.2, 95% *CI*: − 0.7, 1.0; *p* = 0.68).

**Table 3:** Intent-to-treat analysis comparing OMIE SSPs to Control SSPs OEND implementation effectiveness

	**Control SSPs**		**OMIE SSPs**				
**Outcomes**	***n***	**Mean (sd)**	***n***	**Mean (sd)**	**Incidence rate ratio**	**95% *CI***	***p*-value**
Number of people receiving naloxone^a^	49	0.4 (0.3)	45	0.9 (1.4)	2.15	(1.42, 3.25)	< 0.01
Number of naloxone doses distributed ±	50	1.3 (1.2)	46	2.5 (2.6)	1.97^a^	(1.18, 3.30)	0.01
Number of OEND best practices implemented!	51	12.9 (2.1)	51	13.1 (2.2)	0.2	(− 0.7, 1.0)	0.68

*CI* Confidence interval, *sd* Standard deviation

^a^Adjusted for baseline rate due to differences at baseline; 88 SSPs include in final mode. ± 94 SSPs included in final model. !102 SSPs included in final analysis

We considered whether there was a difference in study outcomes among OMIE SSPs that completed 10 + or < 10 sessions, compared to Control SSPs (Table 4). The average rate of naloxone doses distributed was greater among SSPs completing 10 + sessions (*IRR* 2.94, 95% *CI*: 1.67, 5.20; *p* < 0.001) and was approximately the same among SSPs that completed < 10 sessions (*IRR* 0.96, 95% *CI*: 0.51, 1.79; *p* = 0.90), compared to Control SSPs. Similarly, SSPs completing 10 + sessions had a greater rate of participants receiving naloxone per contact compared to Control SSPs (*IRR* 2.65, 95% *CI*: 1.66, 4.22; *p* < 0.001), whereas the rate was only modestly larger among those completing < 10 sessions (*IRR* 1.32, 95% *CI*: 0.76, 2.29; *p* = 0.33). We observed similar findings when we considered whether there was a difference in study outcomes among OMIE SSPs that received 12 + h or < 12 h of support, compared to Control SSPs. We did not observe any statistically significance differences between the number of sessions or hours for SSPs and the number of OEND best practices implemented. No harms were reported from SSPs enrolled in the trial.

**Table 4:** Comparing OMIE SSPs dose to Control SSPs regarding OEND implementation effectiveness

	**Number of people receiving naloxone**		**Number of naloxone doses distributed**		**Number of OEND best practices implemented**	
**Sessions**	**IRR (95% *CI*)**	***p*-value**	**IRR (95% *CI*)**	***p*-value**	**Mean difference** **(95% *CI*)**	***p*-value**
Control SSPs	-		-		-	
OMIE SSPs: < 10 sessions	1.32 (0.76–2.29)	0.33	0.96 (0.51–1.79)	0.90	0.10 (− 1.09, 1.29)	0.87
OMIE SSPs: 10 + sessions	2.65 (1.66–4.22)	< 0.001	2.94 (1.67–5.20)	< 0.001	0.22 (− 0.75, 1.19)	0.66
**Hours**	**IRR (95% *CI*)**	***p*-value**	**IRR (95% *CI*)**	***p*-value**	**Mean difference** **(95% *CI*)**	***p*-value**
Control SSPs	-		-		-	
OMIE SSPs: < 12 h	1.52 (0.91–2.55)	0.11	0.99 (0.56–1.75)	0.90	0.02 (− 1.04, 1.08)	0.97
OMIE SSPs: 12 + h	2.69 (1.65–4.41)	< 0.001	3.02 (1.72–5.29)	< 0.001	0.33 (− 0.72, 1.38)	0.54

## Discussion

We conducted a randomized controlled trial to understand whether the multicomponent OMIE approach improved OEND implementation effectiveness within 105 SSPs located throughout the United States. Compared to control SSPs, SSPs randomized to receive the OMIE approach reported a significant and meaningful increase in the number of people receiving naloxone and the number of naloxone doses distributed. These findings are particularly important as prior studies have directly linked higher levels of these two outcomes with reductions in opioid overdose mortality at the population level [17, 47, 48]. The multifaceted OMIE approach was modelled after the evidence-based ISF strategy [44, 51], and our findings contribute to the growing evidence base that supports facilitation-based implementation strategies [28–39]. These findings are particularly encouraging given SSPs typically face substantial external barriers such as lack of adequate funding, fluctuating costs of naloxone, unsupportive legal environments, and community opposition or harassment which can restrict services [23]. Despite these barriers, our findings show that the OMIE approach can yield substantial and significant improvements in aspects of implementation effectiveness for naloxone.

In addition to the results of our intention to treat analysis, we observed a threshold effect where SSPs participating in 10 + sessions or receiving 12 + h had greater effect sizes with regard to the number of people given naloxone and the number of naloxone doses distributed. SSPs participating in fewer than 10 sessions or receiving less than 12 h did not experience significant changes in either the number of people receiving naloxone or the number of naloxone doses distributed. All OMIE SSPs were offered the ability to meet monthly with facilitators. Therefore, it could be that SSPs that were unable able to achieve the 10-session or 12-h threshold had less organizational capacity to engage with our approach. If an SSP does not have sufficient levels of funding or human resources, the challenges around creating time to reflect and plan intentionally with regard to improving implementation effectiveness could be difficult to surmount. Future initiatives should explore the resource needs for SSPs to engage with similar strategies and examine the intersection of improving financial resources for SSPs along with the OMIE approach [52, 53].

We did not observe significant or meaningful increases among OMIE SSPs regarding implementation quality, as measured by OEND best practice adoption. It is important to consider these findings in light of several factors. SSPs are comprised of a variety of organizational types, have various service delivery models, and have access to varying financial and human resources. Considering best practice implementation within and the measurement of implementation quality across this heterogeneous SSP landscape posed challenges. In addition, facilitators relied on distributing existing educational materials and resources as opposed to developing new resources, which could have been impactful during COVID-19. In many instances, SSPs’ external and internal setting constrained meaningful improvements in OEND best practice implementation. For example, our approach did not address the external contextual dynamics, such as funding availability, legal environment, or community harassment. Additionally, our approach could not shift staffing structures, budget size, or hiring polices within programs. With an average of 12 out of 19 best practices adopted at the onset of the trial, it is also important to remember that baseline levels were relatively high, and the remaining best practices could have been more challenging to implement during the COVID-19 pandemic when face-to-face interaction was limited.

Our findings must also be considered in the context of the emergence of the COVID-19 pandemic during the trial. With the onset of the COVID-19, organizational stress faced by SSPs was significant. Some organizations closed, while other SSPs quickly pivoted to service delivery modalities that involved less person-to-person contact. For our trial, we paused recruitment for 3 months as the initial phases of COVID-19 unfolded. We maintained contact with OMIE SSPs, allowing sessions to focus on understanding new challenges SSPs faced with a changing external context. As programs were adapting during the first few months of COVID-19, we relied primarily on email communication. In addition, specific best practice domains (i.e., “staff training and support”) [43], which had the most opportunity for improvement from baseline levels, could have been the most challenging domains to improve when in-person interaction was limited. As such, COVID-19 may have compromised SSPs’ abilities to improve some aspects of quality. Further, cost of operating programs likely increased with expenses due to personal protection equipment, ventilation, and shields to reduce contact between staff and participants, and the cost of naloxone increased during this period [54, 55]. Given the evidence base showing higher levels of naloxone distribution yield reductions in overdose mortality [17, 47, 48], SSPs may have prioritized making improvements with their consistency (i.e., number of people receiving naloxone, number of naloxone doses distribute) amid the intersecting overdose and COVID-19 crises. Nevertheless, future initiatives should consider additional strategies, such as developing educational materials, as potential adjuncts that might meaningfully improve implementation of newly defined best practices.

The generalizability of our findings requires careful consideration. First, this was a nationally based study of SSPs randomized to two study arms. As such, it follows logically that these findings would extend to SSPs meeting our inclusion criteria. It is also encouraging that budget levels of SSPs enrolled in the trial mirror budget levels from nationally representative samples of SSPs [53]. However, more caution should be applied when considering other evidence-based interventions that are common within SSPs, such as smoking supplies, drug checking, or clinical care as these are likely to be influenced by other external factors.

A noteworthy limitation with our trial is the reliance on self-report for the number of naloxone doses distributed, number of people receiving naloxone, and best practice implementation. Given this trial enrolled 105 SSPs across the US and its territories, it was not practical for the study team to visit each SSP and verify these metrics. However, SSPs routinely document naloxone distribution metrics to report to funders. We focused on the past 3 months of naloxone distribution to maximize the possibility of accurate records, and we also have no reason to believe reporting bias would differ by study arm. Yet, there is a possibility that we could have observed a different relationship if we could have accurately captured a longer timeframe. Furthermore, self-report of best practice implementation could have yielded socially desirable responses from programs or characterization of ideal scenarios as opposed to actual implementation experiences, which would bias our results toward the null. It also remains possible that our findings may not extend to SSPs experiencing a combination of outer and inner setting factors that constrain SSP services (e.g., limited financial resources, unsupportive legal environments, and high levels of community harassment).

## Conclusions

In conclusion, we found evidence supporting the effectiveness of the multifaceted OMIE approach increasing naloxone distribution from SSPs. The OMIE approach was found to be effective despite substantial external shocks (i.e., impacts of COVID-19) during the trial. Our findings have major implications for addressing the overdose crisis, which has continued unabated for decades. Sustained funding from federal agencies and state health departments to support national networks and coalitions to employ OMIE practitioners could significantly improve naloxone distribution from SSPs. Organizations with the mission of capacity building and technical assistance within harm reduction should consider adding this approach. In addition, given our approach did not have a significant impact on best practice implementation, which was relatively high in both conditions, future efforts should seek to add other adjuncts that could assist with advancing implementation quality.

### Supplementary Information

**Additional file 1: Appendix A.** Best practices implementation guide.

## Data Availability

Data will be made available upon reasonable request.

## Abbreviations

ATTC: Addiction Technology Transfer Center
CDC: Centers for Disease Control and Prevention
ISF: Implementation and sustainment facilitation
NSSSP: National survey of syringe services programs
OEND: Overdose education and naloxone distribution
OMIE: Organize and mobilize for implementation effectiveness
SSP: Syringe services program
US: United States
