# Supplementary material for: Organize and mobilize for implementation effectiveness to improve overdose education and naloxone distribution from syringe services programs: a randomized controlled trial
Source: Implement Sci. 2024 Feb 28;19:22. doi: 10.1186/s13012-024-01354-y (PMC10900734; doi:10.1186/s13012-024-01354-y)
Supplement: Supplementary file 1 — Additional file 1: Appendix A. Best practices implementation guide. [file 13012_2024_1354_MOESM1_ESM.pdf]

Overdose Education and

NALOXONE

Distribution (OEND)

BEST PRACTICES

for Syringe Services Programs (SSP)

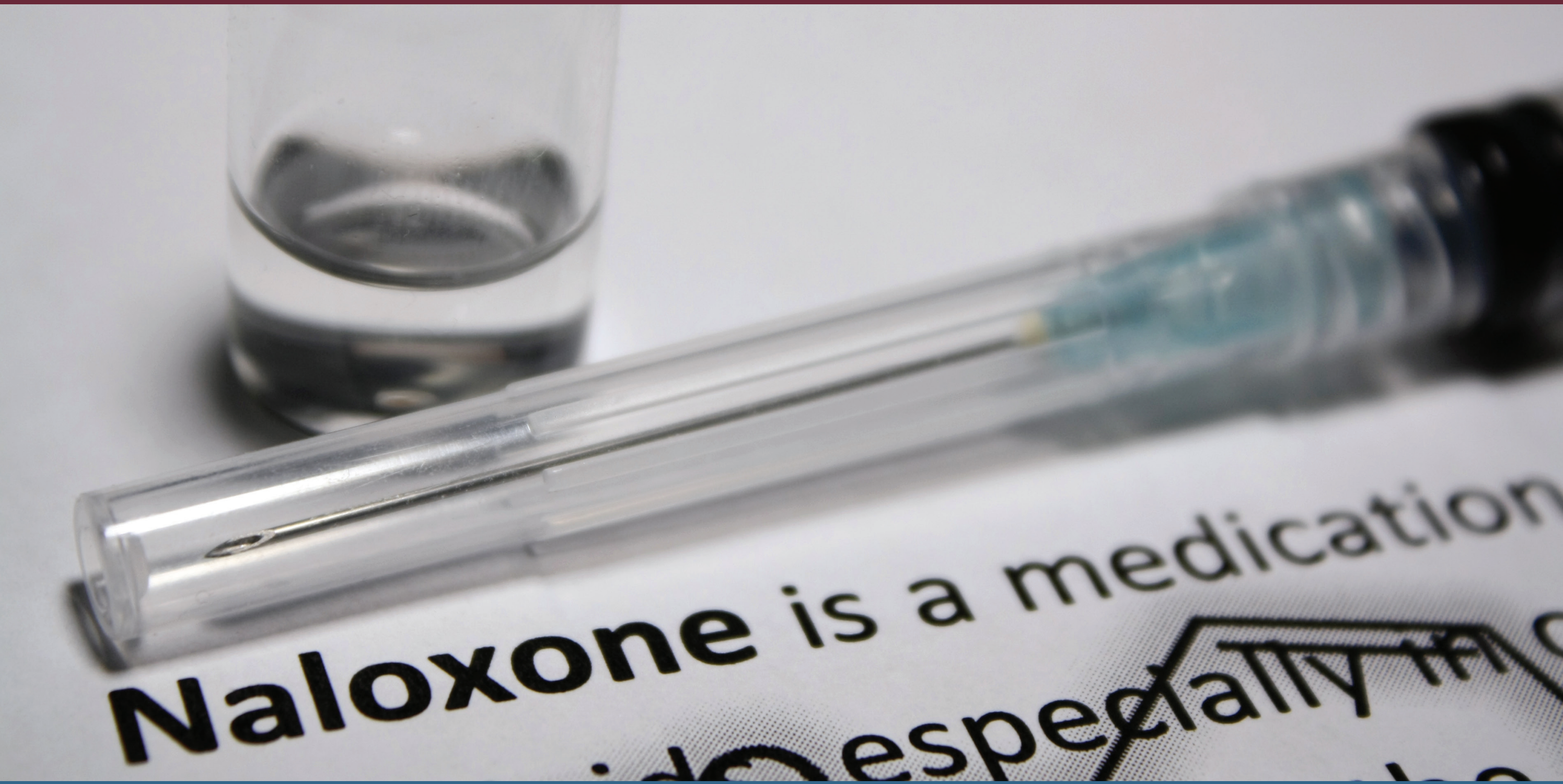

Introduction

Syringe services programs (SSPs) have pioneered public health efforts such as HIV and HCV prevention for people who inject drugs. SSPs have also led efforts for overdose education and naloxone distribution (OEND) programming.[1-3]

In 2001, the first SSP OEND program in the United States was developed at the Chicago Recovery Alliance.[4] Since then, OEND programs have been integrated into SSPs in many areas of country. SSPs are ideal places for OEND programs because staff are experts at providing culturally appropriate services designed to reach persons at high risk for either experiencing or observing an opioid overdose. However, since 2001 some organizations have implemented OEND programs despite legal and regulatory challenges, inadequate resources, and political and community opposition. In spite of these potential barriers, organizations have adapted and developed practices that maximize the reach and impact of their OEND programs and support their staff.

Now, with nearly 20 years of OEND implementation experience, we are documenting the best practices of OEND programming within SSPs to help programs maximize their impact on the communities they serve.

Our Approach

We collaborated with the Harm Reduction Coalition to develop an initial list of best practices for OEND program delivery based out of SSPs. We then gathered input from nearly 30 individuals with expertise in OEND programming throughout the United States, including

- **persons who use drugs,**
- **syringe access program staff and volunteers,**
- **community educators,**
- **harm reduction activists,**
- **researchers, and**
- **representatives from public health departments.**

We first interviewed each expert to get feedback about the best practices and a brief description of them. We then followed up over email to further refine the following best practices.

Best Practices for Overdose Education and Naloxone Distribution

We know that what a best practice looks like in action may differ by the OEND program, by the region of the country where it is located, and by each unique community. A program's ability to use best practices is highly connected to its access to resources and to the unique barriers it faces in delivering services. These best practices include various elements of OEND programming, which we have organized into four areas:

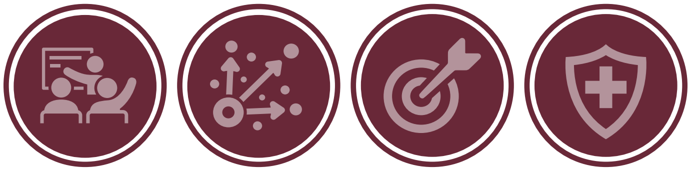

Staff training and support | **Naloxone saturation and supply** | **Culturally appropriate services** | **Grounded in harm reduction**

Staff Training and Support

☐ Proactive Engagement – SSP staff and/or volunteers proactively ask participants if they would like overdose education and naloxone.

Multiple factors can prevent SSP participants from accessing naloxone when it is available, including

- not knowing or forgetting that the SSP offers it,
- being new to the SSP or not yet trusting the program or staff,
- feeling uncomfortable or rushed at a chaotic or busy site, and
- and being in a hurry and not knowing that naloxone can be accessed quickly.

Proactively engaging participants around OEND lets them know that naloxone is readily available and shows that it is a priority of the program to ensure that participants have the knowledge and supplies they need to keep themselves and their community alive.

☐ Needs-based Training – OEND training can be completed in as little as 5 to 10 minutes and follows the participant's needs.

OEND training with a set time frame and curriculum can be a barrier to SSP participants accessing naloxone. Instead, to support the experience and autonomy of SSP participants,

- OEND training can be tailored to participants' specific desires and needs,
- shorter training can be offered to participants who are in a rush or already feel confident in their knowledge and ability around opioid overdose response, and
- longer training can be offered to participants who desire more knowledge.

Also, research has shown that OEND training lasting 5 to 10 minutes is sufficient to improve participants' comfort level and ability to recognize and respond to an opioid overdose.[5]

☐ Support for Vicarious Trauma – SSPs provide support for overdose prevention educators to address experiences of vicarious trauma.

Vicarious trauma is what an individual can experience by hearing about another person's traumatic experiences or witnessing how their trauma has affected them. Over time, this can lead to changes in psychological, physical, and spiritual well-being. It also can make working difficult and unpleasant.[6]

Providing program staff training to help identify and develop practices that support resiliency and offering support around processing vicarious trauma can make working with highly traumatized individuals safer and more sustainable.

☐ On-site Overdose Protocol Established – SSPs have an onsite overdose protocol, and staff/volunteers are trained to respond to an overdose in the course of providing services.

Having an overdose response protocol that staff/volunteers are trained to use can address anxiety that someone may fatally overdose on-site. Also, having this protocol lets participants know that the program prioritizes their safety and well-being, even when they may be breaking program rules.

☐ Support for Burnout – SSPs provide support for overdose prevention educators to address burnout.

Burnout is the feeling of physical and emotional exhaustion caused by stress from working with people under difficult or demanding conditions. Burnout can be characterized by

- compassion fatigue,
- difficulty maintaining professional boundaries, and
- increased susceptibility to vicarious trauma.[7]

Experiencing burnout can be a normal part of doing stressful and emotionally intense work. Providing program staff with training to identify and develop practices that support resiliency and offering support around processing experiences that can lead to burnout can help make physically and emotionally taxing work safer and more sustainable.

☐ Training of Trainers – Overdose prevention educators are trained in the following areas:

- Engagement, counseling, and listening skills
- Delivering health education for safer drug use, overdose prevention/response, and naloxone administration
- Working with participants to develop personal overdose prevention plans
- Supporting participants around experiences of witnessing overdoses and administering naloxone
- Providing referrals to health, substance use, and social services

Naloxone Saturation and Supply

☐ Needs-based Naloxone Distribution – Naloxone distribution is based on participants' needs and the needs of their community.

Factors other than a program participant's individual risk for experiencing or witnessing an opioid overdose can affect how much naloxone they need at the time they visit an OEND program. For example, participants

- may be accessing supplies and education on behalf of their household, friends, encampment, or other forms of community;
- may not want to or be able to access OEND services frequently; or
- may be in contact with persons at risk for experiencing or witnessing an opioid overdose but who are not engaged in OEND services.

Participants should be able to request and be provided with as much naloxone as they need for themselves and their broader community. This includes secondary naloxone distribution, where a participant obtains naloxone specifically to distribute directly to others in their social networks. This type of needs-based naloxone distribution recognizes that individuals know their needs and those of their community and directly supports them in meeting those needs.

☐ Naloxone Is Accessible – Overdose education training and naloxone distribution are provided at all SSP sites and during community-based outreach.

Providing naloxone at all SSP sites and during outreach maximizes the opportunities for SSP participants to engage in OEND services. More opportunities for naloxone access also can relieve pressure on staff and volunteers during days and/or times with a high volume of participants, when there is less time available for each participant.

☐ Sufficient Naloxone Supply – Naloxone inventory is accessible to SSP staff/volunteers, and the OEND program has enough naloxone to not run out or to need to ration distribution for the next 3 months.

Keeping an excess of naloxone on hand and accessible to SSP staff can enable service continuity if there were to be a natural disaster, a decrease in funding, or some other event that disrupts naloxone supply. Knowing other naloxone access points in the surrounding community –community-based programs, local health departments, pharmacies, etc. – is critical to being able to link people to other naloxone sources if there is a shortage in an SSPs naloxone supply.

☐ Naloxone Saturation – Annually, SSPs distribute 20 times or more the number of naloxone doses as the number of opioid overdose deaths in the previous year (or for the most recent year overdose death data are available).

For example, if the county where the SSP operates had 100 opioid overdose deaths in the previous year, the goal for the current year would be to distribute at least 2,000 doses of naloxone to participants. One study showed that programs that reached or exceeded 20 times the number of naloxone doses as the number of opioid overdose deaths in the previous year observed a 62% reduction in opioid overdose deaths in the community.[8]

☐ Option to Choose Naloxone Administration Modality – Participants can choose intranasal or intramuscular naloxone, based on their preferences.

Several factors can influence a person’s preference for which form of naloxone they prefer to use, including

- local paraphernalia laws,
- the stigma surrounding syringe use,
- previous experience using intranasal or intramuscular naloxone, and
- discomfort injecting someone else.

Offering both intramuscular and intranasal forms of naloxone increases the chances that program participants will feel comfortable both carrying and using their naloxone.

Culturally Appropriate Services

☐ **Involvement of People Who Use Drugs – People who use drugs and who are active SSP participants deliver overdose prevention education, distribute naloxone, contribute to naloxone programming, and provide oversight of program activities.**

Involving people who use drugs in program activities

- ensures that OEND programming is community informed and community led,
- enables programs to benefit from the expertise of people who are most impacted,
- increases the likelihood that the program serves the community and people with the highest need.

☐ **Lay Person Naloxone Team – SSPs use nonmedical staff/volunteers to provide overdose prevention education and naloxone distribution.**

Using lay people rather than medical staff to provide OEND services reduces the burden on programs to find and pay a medical provider who will work as often as is needed. Also, program participants often find lay people who staff the SSP with whom they are already familiar to be more approachable and trustworthy than medical providers.

☐ **Overdose Response Information and Education Materials Offered – Educational materials about overdose risk and response, such as pamphlets, posters, palm cards, and/or web-based resources, are available to SSP participants.**

Providing educational materials and web-based resources to OEND program participants

- gives them tools to review information and learn independently,
- allows them to access information as they need it, and
- enables them to pass materials and resources on to others in their social networks who may not be engaged in OEND services.

☐ **Outreach and Marketing Conducted – SSPs publicize naloxone programming by distributing information through flyers, pamphlets, posters, and social media; building community partnerships; and conducting community outreach.**

Having a presence in the community is a strategy that seeks to reach people at risk for experiencing or witnessing opioid overdose in places where they hang out, access services, and spend their time. This approach increases the chances that people who are at risk will learn of and access OEND services.

Grounded in Harm Reduction

☐ OEND Program Is Grounded in Harm Reduction Principles – SSP staff/volunteers are trained and supported to offer services grounded in harm reduction principles.

According to Harm Reduction International, harm reduction is a set of evidence-based practices that minimizes the negative impact of drug use and ineffective drug policies. Harm reduction is grounded in principles that protect human rights and improve public health. It incorporates a wide array of approaches designed to meet people where they are and to offer opportunities for people to improve their health and well-being.

Harm Reduction Coalition has a set of 8 principles that are central to harm reduction practice. These principles can be found here: https://harmreduction.org/about-us/principles-of-harm-reduction/

☐ Low Threshold Services – SSPs offer walk-in services without the need to make appointments.

Much like offering OEND services during all SSP site hours and community-based outreach, offering walk-in services without the need to make appointments allows program participants to access naloxone at their convenience without needing to schedule ahead and arrive on time for an appointment. This approach also enables program staff to offer naloxone to participants in the moment, rather than at a later time or date.

☐ Naloxone at No Cost – Naloxone is distributed by SSPs and is free of cost.

People accessing naloxone through pharmacies may face significant financial barriers to getting naloxone. Even when certain forms of naloxone may be covered by health insurance or stipends provided by regional overdose prevention initiatives, lack of pharmacists' education on how these people can benefit from naloxone may prevent them from offering it.

SSPs that offer naloxone on-site and free of cost ensures that people who are at risk of experiencing or witnessing an opioid overdose have access to naloxone regardless of their ability to pay for it.

☐ Anonymous Service Delivery – Participants are not required to provide personal information or identification to receive naloxone.

There are many reasons why OEND program participants may want to remain anonymous when accessing services. Respecting participants' desire to remain anonymous—even if a program normally requires participants to share personally identifiable information to access services—shows that the program respects participants' privacy and prioritizes participants' health and well-being.

☐ Only Essential Data are Collected – SSPs only collect essential information from participants for program improvement, reporting, or advocacy.

Collecting data on the identity and experiences of OEND program participants can be time consuming, invasive, and possibly deter some participants from accessing OEND services. But these factors should be weighed against the benefits that collecting data can provide for improving the program, reporting requirements to receive funding, and conducting advocacy work that can build a stronger OEND program response for the community.

References

1. Enteen L, Bauer J, McLean R, Wheeler E, Huriaux E, Kral AH, et al. Overdose Prevention and Naloxone Prescription for Opioid Users in San Francisco. J Urban Health. 2010;87(6):931-41.

2. Doe-Simkins M, Walley AY, Epstein A, Moyer P. Saved by the Nose: Bystander-Administered Intranasal Naloxone Hydrochloride for Opioid Overdose. American journal of public health. 2009;99(5):788-91.

3. Wheeler E, Davidson PJ, Jones TS, Irwin KS. Community-Based Opioid Overdose Prevention Programs Providing Naloxone — United States, 2010. MMWR Morb Mortal Wkly Rep. 2012;61(6):101-15.

4. Maxwell S, Bigg D, Stanczykiewicz K, Carlberg-Racich S. Prescribing naloxone to actively injecting heroin users: a program to reduce heroin overdose deaths. Journal of addictive diseases. 2006;25(3):89-96.

5. Behar E, Santos GM, Wheeler E, Rowe C, Coffin PO. Brief overdose education is sufficient for naloxone distribution to opioid users. Drug and alcohol dependence. 2015;148:209-12.

6. Pearlman LA, McKay L. Understanding and Addressing Vicarious Trauma. Available at: https://headington-instituteorg/files/vtmoduletemplate2_ready_v2_85791pdf.

7. Gandi JC, Wai PS, Karick H, Dagona ZK. The role of stress and level of burnout in job performance among nurses. Ment Health Fam Med. 2011;8(3):181-94.

8. Bird SM, Parmar MK, Strang J. Take-home naloxone to prevent fatalities from opiate-overdose: Protocol for Scotland’s public health policy evaluation, and a new measure to assess impact. Drugs (Abingdon Engl). 2015;22(1):66-76.

Overdose Education and
NALOXONE
Distribution (OEND)

BEST PRACTICES
for Syringe Services Programs (SSP)

Staff Training and Support

- [ ] **Proactive Engagement**
SSP staff and/or volunteers proactively ask participants if they would like training and/or need naloxone.

- [ ] **Needs-based Training**
OEND training can be completed in as little as 5 to 10 minutes and follows the participant's needs.

- [ ] **Support for Vicarious Trauma**
SSPs provide support for overdose prevention educators to address experiences of vicarious trauma.

- [ ] **Support for Burnout**
SSPs provide support for overdose prevention educators to address burnout.

- [ ] **On-site Overdose Protocol Established**
SSPs have an onsite overdose protocol, and staff/volunteers are trained to respond to an overdose in the course of providing services.

- [ ] **Training of Trainers**
Are trained in the following areas:
  - Engagement, counseling, and listening skills
  - Delivering health education for safer drug use, overdose prevention/response, and naloxone administration
  - Working with participants to develop personal overdose prevention plans
  - Supporting participants around experiences of witnessing overdoses and administering naloxone
  - Providing referrals to health, substance use, and social services

Resources/Naloxone Policy Saturation of Services

- [ ] **Needs-based Naloxone Distribution**
Naloxone distribution is based on participants' needs and the needs of their community.

- [ ] **Naloxone is Accessible**
Overdose education training and naloxone distribution are provided at all SSP sites and during community-based outreach.

- [ ] **Sufficient Naloxone Supply**
Naloxone inventory is accessible to SSP staff/volunteers, and the OEND program has enough naloxone to not run out or to need to ration distribution for the next 3 months.

- [ ] **Naloxone Saturation**
Annually, SSPs distribute 20 times or more the number of naloxone doses as the number of opioid overdose deaths in the previous year (or for the most recent year overdose death data are available).

- [ ] **Option to Choose Naloxone Administration Modality**
Participants can choose intranasal or intramuscular naloxone, based on their preferences.

Culturally Appropriate Services

- [ ] **Involvement of People Who Use Drugs**
People who use drugs and who are active OEND program participants deliver overdose prevention education, distribute naloxone, contribute to naloxone programming, and provide oversight of program activities.

- [ ] **Lay Person Naloxone Team**
SSPs use nonmedical staff/volunteers to provide overdose prevention education and naloxone distribution.

- [ ] **Overdose Response Information and Education Materials Offered**
Educational materials about overdose risk and response, such as pamphlets, posters, palm cards, and/or web-based resources, are available to SSP participants.

- [ ] **Outreach and Marketing Conducted**
SSPs publicize naloxone programming by distributing information through flyers, pamphlets, posters, and social media; building community partnerships; and conducting community outreach.

Grounded in Harm Reduction

- [ ] **OEND Program Is Grounded in Harm Reduction Principles**
SSP staff/volunteers are trained and supported to offer services grounded in harm reduction principles.

- [ ] **Low Threshold Services**
SSPs offer walk-in services without the need to make appointments.

- [ ] **Naloxone at No Cost**
Naloxone is distributed by SSPs and is free of cost.

- [ ] **Anonymous Service Delivery**
Participants are not required to provide personal information or identification to receive naloxone.

- [ ] **Only Essential Data are Collected**
SSPs only collect essential information from participants for program improvement, reporting, or advocacy.
